# Characterization of neutralizing epitopes within the major capsid protein of human papillomavirus type 33

**DOI:** 10.1186/1743-422X-3-83

**Published:** 2006-10-02

**Authors:** Stefanie D Roth, Martin Sapp, Rolf E Streeck, Hans-Christoph Selinka

**Affiliations:** 1 Institute for Medical Microbiology, Johannes Gutenberg-University 55101 Mainz, Germany; 2 Center for Molecular and Tumor Virology, Louisiana State University Health Sciences Center, 1501 Kings Highway, Shreveport, LA 71130, USA; 3 Feist Weiller Cancer Center, Louisiana State University Health Sciences Center, 1501 Kings Highway, Shreveport, LA 71130, USA; 4 Department of Microbiology and Immunology, Louisiana State University Health Sciences Center, 1501 Kings Highway, Shreveport, LA 71130, USA

## Abstract

**Background:**

Infections with papillomaviruses induce type-specific immune responses, mainly directed against the major capsid protein, L1. Based on the propensity of the L1 protein to self-assemble into virus-like particles (VLPs), type-specific vaccines have already been developed. In order to generate vaccines that target a broader spectrum of HPV types, extended knowledge of neutralizing epitopes is required. Despite the association of human papillomavirus type 33 (HPV33) with cervical carcinomas, fine mapping of neutralizing conformational epitopes on HPV33 has not been reported yet. By loop swapping between HPV33 and HPV16 capsid proteins, we have identified amino acid sequences critical for the binding of conformation-dependent type-specific neutralizing antibodies to surface-exposed hyper variable loops of HPV33 capsid protein L1.

**Results:**

Reactivities of monoclonal antibodies (mAbs) H33.B6, H33.E12, H33.J3 and H16.56E with HPV16:33 and HPV33:16 hybrid L1 VLPs revealed the complex structures of their conformational epitopes as well as the major residues contributing to their binding sites. Whereas the epitope of mAb H33.J3 was determined by amino acids (aa) 51–58 in the BC loop of HPV33 L1, sequences of at least two hyper variable loops, DE (aa 132–140) and FGb (aa 282–291), were found to be essential for binding of H33.B6. The epitope of H33.E12 was even more complex, requiring sequences of the FGa loop (aa 260–270), in addition to loops DE and FGb.

**Conclusion:**

These data demonstrate that neutralizing epitopes in HPV33 L1 are mainly located on the tip of the capsomere and that several hyper variable loops contribute to form these conformational epitopes. Knowledge of the antigenic structure of HPV is crucial for designing hybrid particles as a basis for intertypic HPV vaccines.

## Background

Human papillomavirus (HPV) infection is the obligate first step in the development of cervical cancer [1]. However, of the more than 100 types of HPV, only 15 so-called high risk types, most commonly types 16, 18, 31, 33, 39, 45, 52, and 58, account for at least 95% of HPV-induced cervical cancer [2,3]. Vaccination against these high risk types seems to be the most feasible prevention for cervical cancer. Indeed, clinical trials have shown prophylactic HPV vaccines to be effective against HPV infection, cervical intraepithelial neoplasia (CIN), and genital warts, but protection is type-specific and the currently developed vaccines target only a few types [4-6]. These vaccines are based on papillomavirus-like particles (VLPs) composed of the major capsid protein, L1. The L1 protein self assembles into VLPs when expressed at high levels in eukaryotic or insect cells [7-10]. VLPs are composed of 360 copies of L1 protein organized into 72 pentamers, so called capsomeres, to form particles which are immunologically indistinguishable from native virions. Experimentally induced VLP antisera have been shown to be mostly type-specific with respect to neutralization [11-13]. Minor cross-neutralization has been observed only between closely related HPV types, e.g. HPV6 and 11, HPV18 and 45, or HPV16 and 31 [14-16]. Structure analysis has revealed the presence of several hyper variable loops on the outer surface of the capsid [17]. With a few exceptions, all HPV-neutralizing monoclonal antibodies analyzed so far are type-specific and recognize conformational epitopes within surface-exposed hyper variable loops of the major capsid protein L1 [18-21]. Since capsomeres are also potent immunogens for induction of neutralizing antibodies, the formation of these conformational epitopes does not necessarily require capsid assembly [22,23]. In a few cases, cross-neutralizing monoclonal antibodies raised against VLPs in animals that recognize surface-exposed linear epitopes have been described [14,16,21].

A prerequisite for generating vaccines that prevent infection with a broad spectrum of HPV types is extended knowledge of viral determinants provoking common and type-specific immune responses. In the present study, we have fine mapped the binding sites of three neutralizing monoclonal antibodies (H33.B6, H33.E12, and H33.J3) with specificity for the human papillomavirus high risk type 33 (HPV33) by site-directed mutagenesis of surface-exposed amino acids in the major capsid protein L1. Moreover, HPV16:33BC hybrid pseudovirions, formed by HPV16 L1 proteins containing amino acids 51–58 of HPV33 L1 and HPV16 L2, assembled into particles which could be neutralized by both HPV33- and HPV16-specific antibodies, confirming the functional expression of intrinsic and ectopically expressed epitopes.

## Results

### Neutralization of HPV33 pseudovirus infection

**Figure 1:**
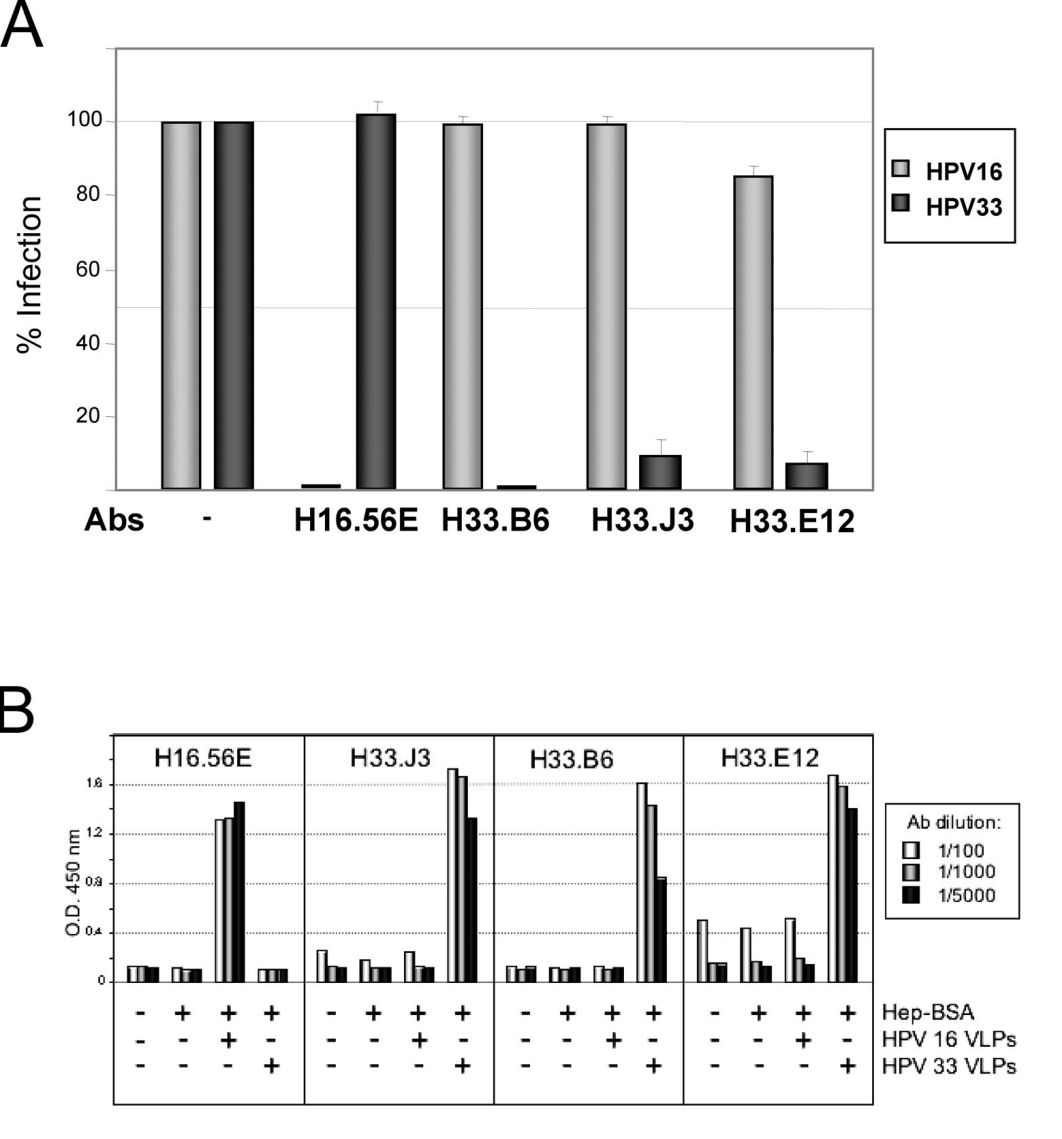
**Type-specificity of HPV-reactive antibodies**. A) Infection of human 293TT cells with HPV16 and HPV33 pseudovirions in the presence of type-specific neutralizing antibodies. Infectious events unaffected by HPV16-specific (mAb H16.56E) or HPV33-specific (mAbs H33.B6, H33.J3) monoclonal antibodies were monitored 72 hrs post infection. B) Interaction of type-specific antibodies with HPV16 and HPV33 virus-like particles (VLPs) in a Heparin-BSA ELISA assay. All three antibodies displayed type-specificity. Although background binding of mAb H33.E12 is significantly increased, specific binding is also restricted to particles of HPV type 33.

Papillomavirus pseudovirions that encapsidate a marker plasmid instead of the viral genome are widely used to study HPV biology and infection, circumventing the difficulties to obtain biochemical quantities of native virions [12,24]. Using such HPV16 and HPV33 pseudovirions, we first determined the neutralizing potential of various HPV-specific antibodies (Fig. 1). Three days post infection with HPV pseudovirions, infection was monitored by the number of cells with green nuclear fluorescence, caused by transmission of a GFP marker gene to the nucleus via the HPV vector. Pseudovirus infection in the presence of the HPV33-specific neutralizing monoclonal antibodies (mAbs H33.B6, H33.J3, and H33.E12) was abolished only with pseudovirions of the respective type. Moreover, we used the recently described mAb H16.56E, generated after immunization with HPV16 VLPs, and also observed type-specific neutralization, demonstrating the validity of this surrogate system for use in testing papillomavirus neutralizing antibodies (Fig. 1A). Binding of these antibodies to conformationally intact HPV VLPs bound to Heparin-BSA-coated Elisa plates confirmed the selective specificity of antibodies H33.J3, H33.B6 and H33.E12 for HPV33 (Fig. 1B). Subsequent experiments were performed to characterize and fine map the epitopes of these HPV33-specific antibodies.

### Characterization of hyper variable regions in HPV33 L1

**Figure 2:**
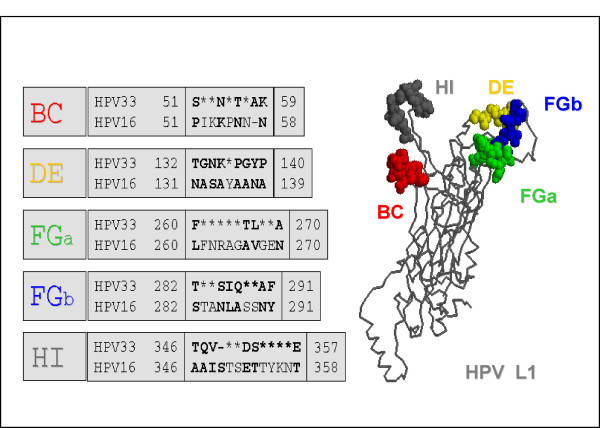
**Determinants of type-specificity**. Alignment of amino acid sequences in surface-exposed loops of capsid proteins L1 of HPV16 and HPV33. Divergent amino acids are listed; identical amino acids are marked by asterisks. On the right, localization of these hyper variable loops in the L1 monomer is shown. Modeling by RasMol was based on the monomeric structure of the HPV16 capsid protein L1.

For various HPV types it has been reported that type-specific monoclonal antibodies primarily reside in surface-exposed hyper variable loops. Our experimental approach for defining residues involved in neutralization of HPV33 by mAbs H33.B6, H33.J3 and H33.E12 was therefore based on the exchange of type-specific loop sequences between the closely related papillomavirus types 16 and 33. Poorly conserved regions in HPV major capsid proteins L1 were identified by sequence alignment and localized by RasMol, based on the coordinates of HPV16 (Pdb file 1DZL). As shown in Fig. 2, 30 divergent amino acids between HPV33 and HPV16 were localized in 4 surface-exposed hyper variable loops, named BC (aa 51–59), DE (aa 132–140), FG (260–291), and HI (346–358), according to the HPV16 L1-structure reported by Chen et al. [17]. In HPV33 L1, the FG loop was found to consist of two separate hyper variable regions, designated in this paper as FGa (260–270) and FGb (282–291) (Fig. 2).

### Functional characterization of HPV33 epitopes by loop substitution

**Figure 3:**
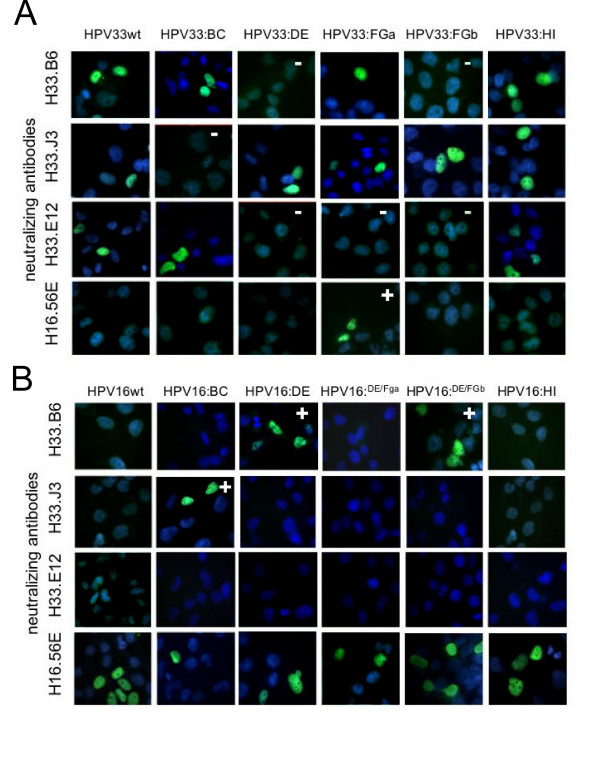
**Epitope mapping of type-specific antibodies**. **A) **Elimination of HPV33-specific epitopes by loop exchanges in capsid protein L1. Recombinant HPV L1 capsid proteins expressed in HUTK^- ^cells were tested by immunofluorescence analysis for the presence of epitopes for antibodies H16.56E, H33.E12, H33.J3 and H33.B6. Loss of reactivity is marked by (-), gain of antibody reactivity by (+). **B) **Functional transfer of HPV33-specific epitopes to HPV 16 by loop swapping, leading to reactivity (+) with the respective HPV33-specific antibody. Note that the correct presentation of corresponding epitopes is also influenced by neighboring loops.

To further characterize the epitopes of HPV33-specific antibodies, hybrid virus-like particles were designed in which type-specific sequences in the major capsid protein L1 of HPV33 were replaced by corresponding amino acids of HPV16, eliminating the putative epitopes. Vice versa, HPV33-specific sequences were introduced into HPV16 L1 for ectopic expression. Ten different hybrid L1 proteins (HPV33:16BC; HPV33:16DE; HPV33:16FGa; HPV33:16FGb; HPV33:16HI; HPV16:33BC; HPV16:33DE; HPV16:33DE/FGa, HPV16:33DE/FGb, and HPV16:33HI) were constructed and expressed in HUTK^-^-143B cells. Western blot analysis revealed that all hybrid proteins were expressed at similar levels (data not shown). Binding of monoclonal antibodies to hybrid L1 protein was first tested by immunofluorescence under non-denaturing conditions (Fig. 3). Reactivity of H33.J3 with hybrid particles was lost by exchanging the BC loop but was retained after replacement of the other loops (Fig. 3A). This suggests that the BC loop is the binding site for H33.J3 and that exchange of neighboring surface loops results in conformationally intact L1 assemblies. HPV16 L1 hybrid particles became reactive with this antibody when the HPV33 BC loop, but not the DE, FG, and HI loops, were ectopically expressed on HPV16 (Fig 3B). Reactivity of H16:56E with HPV16:33BC was retained, suggesting that this antibody recognizes a different epitope and, in addition, that this hybrid L1 protein also forms conformationally intact assemblies.

The epitope recognized by the H33.B6 antibody was shown to be more complex, as exchange of loops DE or FGb resulted in the loss of reactivity. Vice versa, introduction of both HPV33 loops into HPV16 L1 transferred reactivity of H33.B6 to the HPV16:33DE/FGb hybrid (Fig. 3B). Surprisingly, exchange of the DE loop alone was sufficient to render HPV16:33DE reactive with this antibody. However, the concomitant exchange of the DE and FGa loops abrogated the binding of H33.B6 with HPV16:33DE/FGa. Therefore, without being part of the epitope, the FGa loop has significant influence on the conformation of the DE loop and thus contributes to the conformation recognized by H33.B6.

The monoclonal antibody H33.E12 binding site also displays a high level of complexity. Individual swapping of loops DE, FGa, and FGb results in the loss of binding to HPV33 hybrid L1 proteins (Fig. 3A), whereas the exchange of the BC and HI loops had no effect. In contrast to H33.B6, transfer of individual or two combined HPV33 loops onto HPV16 did not result in the reconstruction of the epitope. Unfortunately, we were not successful in the construction of hybrid 16L1 protein carrying all three HPV33 loops required for binding of H33.E12. Using our HPV16:33 chimeric particles, we could also show that the FGa loop is an important part of the H16.56E epitope, since only HPV33:16FGa particles were recognized by this antibody. Vice versa, the fact that all HPV16:33 chimeras were still recognized by this antibody demonstrates that the H16.56E binding site is not a one-loop epitope but rather formed by discontiguous sequences of the L1 protein.

**Figure 4:**
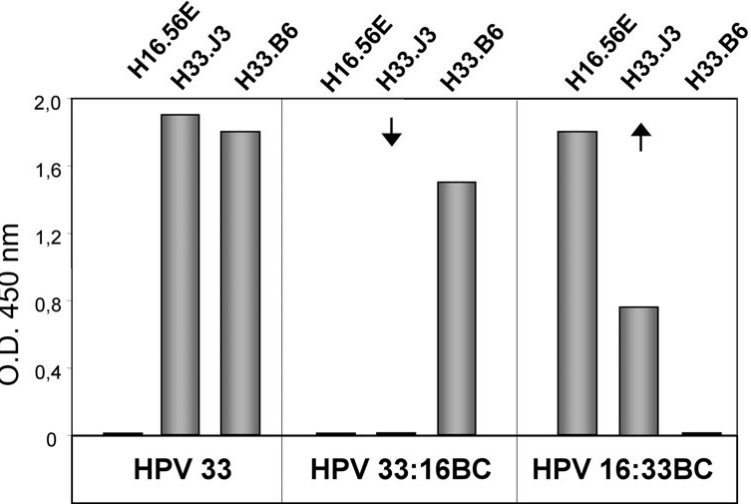
**Heparin-BSA ELISA**. Analysis of epitope expression on wild type (HPV33) and chimeric (HPV33:16BC and HPV16:33BC) VLPs bound to Heparin-coated ELISA plates using type-specific antibodies H16.56E, H33.J3 and H33.B6. Exchange of aa 51–58 (BC-loop of capsid protein L1) results in the loss or gain of reactivity with antibody H33.J3.

To confirm the validity of our immunofluorescence approach for measuring conformation-dependent antibody binding, we generated and purified hybrid HPV33:16BC VLPs, using recombinant vaccinia viruses and HPV16:33BC after transfection of codon-optimized L1. Reactivity of the monoclonal antibodies with VLPs was measured in a heparin-BSA ELISA (Fig. 4). Swap of the BC loop resulted in the loss of reactivity of hybrid HPV33:16BC with H33.J3 and a gain of reactivity with H16:33BC. Binding of H33.B6 and H16.56E were not affected by this exchange and solely dependent on the backbone (33L1 for HPV33:16BC and 16L1 for HPV16:33BC) of the chimeric L1 molecules.

### Neutralization of hybrid pseudoviruses

**Figure 5:**
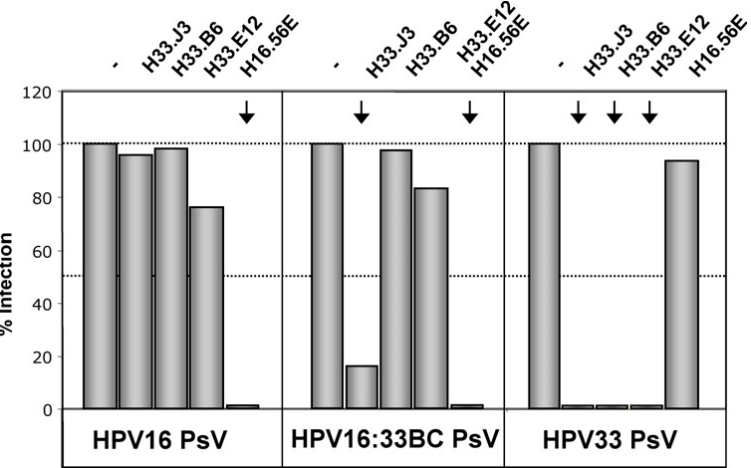
**Neutralization of HPV pseudovirus infection of 293TT cells by type-specific antibodies**. In contrast to wt HPV16 and HPV33 pseudovirions, HPV16:33BC pseudovirions are neutralized by the HPV16-specific H16.56E as well as the HPV33-specific H33.J3 antibodies. Infection was monitored 72 h post infection.

To exemplarily demonstrate that the transfer of HPV33-specific epitopes is functional, hybrid pseudovirions HPV16:33BC were generated that contain the HPV33 BC loop in the context of HPV16, following a published protocol [24]. The mutant was cotransfected with the HPV16 wtL2 expression plasmid and a GFP-expressing marker plasmid to be packaged. The mutant protein efficiently assembled with the L2 protein and the marker plasmid into pseudoviruses that were used in subsequent neutralization assays. As shown in Fig. 5, HPV16:33BC and wt HPV33, but not wt HPV16 pseudovirions, were efficiently neutralized by H33.J3. Hybrid viruses were not neutralized by H33.B6 and H33.E12. These data clearly demonstrate the functional expression of the heterotypic epitope on HPV16.

## Discussion

**Figure 6:**
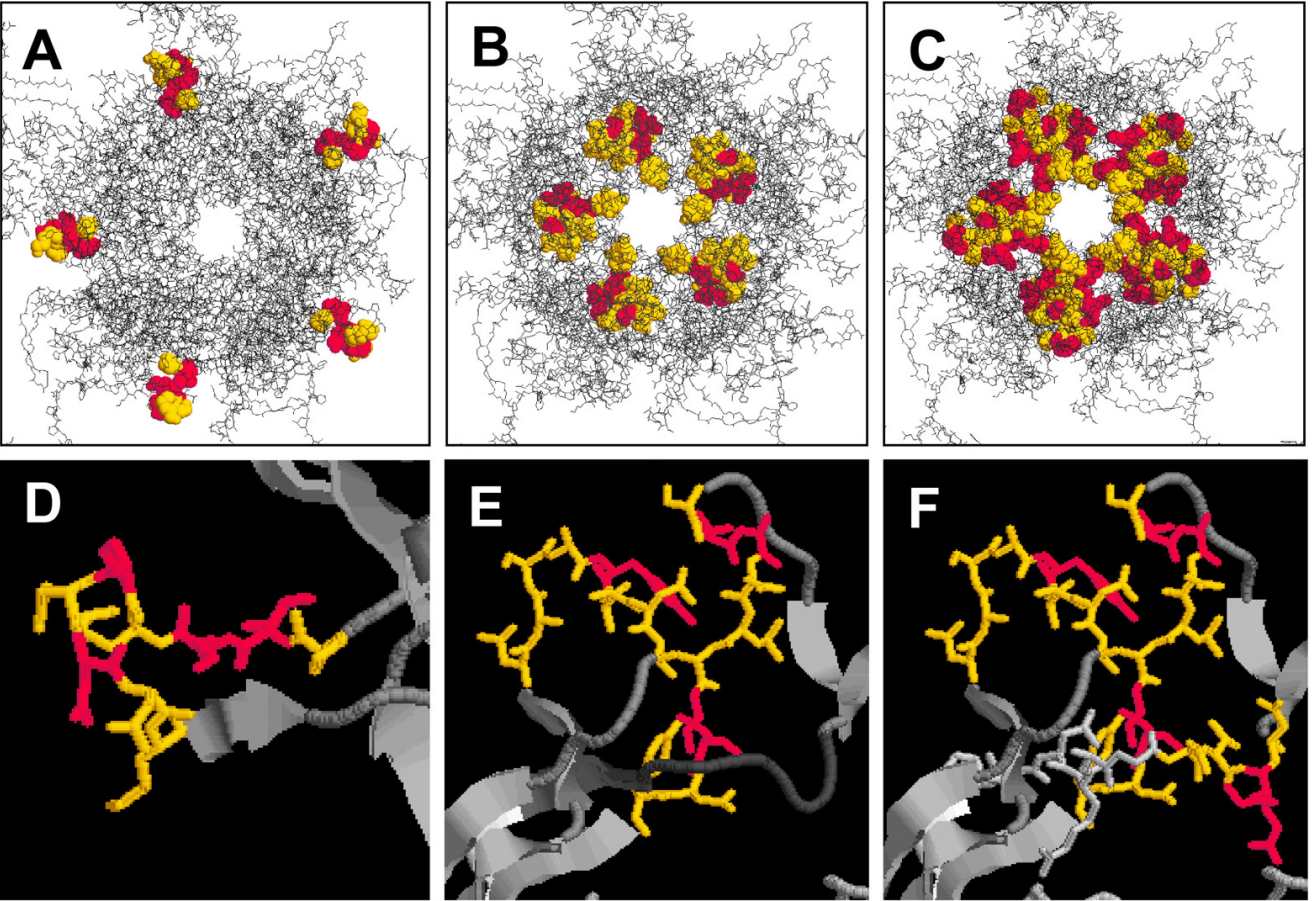
**Epitopes of HPV33-specific antibodies on the pentameric L1 capsomere**. RasMol pictures showing the epitope patterns for mAb H33.J3 **(A)**, mAb H33.B6 **(B) **and mAb H33.E12 **(C)**. Variations in the complexity of the epitopes **(D-F)**, ranging from a single loop (D; H33.J3 epitope), two neighboring loops (E, H33.B6 epitope), to at least three loops (F; H33.E12 epitope). Type-specific amino acids are shown in yellow, conserved amino acids in red).

A variety of neutralizing epitopes are expressed on the capsid surface of human papillomaviruses. So far, neutralizing antibody binding sites for HPV6, 11, 16, 31, and 52 have been mapped to the hyper variable surface loops BC, DE, FG, and HI of the major capsid protein L1 [17,19,20,25-27]. In addition, one neutralizing epitope has been recently identified in the carboxyl-terminal arm of HPV16 (aa 430–450) [28]. The complexity of these epitopes differs considerably among the monoclonal antibodies analyzed so far. We have now demonstrated the involvement of the BC, DE, and FG surface loops of HPV33 L1 in the induction of type-specific immune responses. H33.J3 recognizes a conformation which solely depends on the presence of the BC loop (Fig. 6A, D). This seems to be a rare event, since most epitopes of neutralizing antibodies recognize conformations depending on more than one loop. By swapping BC loops, the binding and neutralization capacity of this HPV33-specific antibody was easily transferable onto HPV16. The H33.J3 epitope is determined by amino acids 51–58 and is located at the vertices of capsomeres. Only very few antibodies specific for HPV6 and 11 have been reported to bind this loop [27], and no HPV high-risk type-specific antibody other than H33.J3 has been mapped to this region so far. This may explain the unique properties of this antibody, which does not interfere with binding of particles to the primary HPV attachment receptor, heparan sulfate proteoglycan, and its characteristic feature to preferentially neutralize cell-bound rather than free pseudoviruses [29].

We demonstrated that a more complex epitope is recognized by H33.B6 (Fig. 6B, E). Both the DE and the FGb loop are necessary for binding. Our data also suggest that the FGa loop contributes to the conformation recognized by H33.B6 without being part of the binding site. This is not surprising since all three loops are in intimate proximity to each other and other monoclonal antibodies have also been shown to be influenced by more than one of these loops [20]. The H33.E12 antibody is dependent on loops DE, FGa, and FGb, since replacement of each of these loops for HPV16 resulted in the loss of reactivity. This defines the H33.E12 binding site as an even more complex epitope (Fig. 6C, F). The previously observed partial cross-reactivity of H33.J3 with HPV45, 58, and 59 [16] is most likely due to the complex binding site of this antibody. However, in most cases, cross-reaction might not be sufficient for cross-protection.

Using the HPV16:33BC chimera in pseudovirus neutralization assays, we have also shown that the BC hyper variable loop swap not only transfers the binding ability of H33.J3 but also the neutralizing capacity to HPV16. This result suggests that it should be possible to generate HPV hybrid particles that elicit an immune response directed to more than one HPV type. Because of the complexity involving loops DE, FG, and also probably HI [20], which all can contribute to the conformational binding site of a given antibody, targeting loops that are clearly separated seems to be more promising. In addition to the BC loop, the carboxyl terminal arm is probably a good candidate for such an approach. Only few antibodies that are directed against these regions, which were obtained after experimental immunization of animals, have been described in the literature so far. This could possibly indicate that these epitopes are not immunodominant. On the other hand, a recent analysis of the humoral immune response induced by natural infection with HPV6 and HPV11 did reveal that all L1 surface loops induced efficient immune responses, and failed to identify any immunodominant epitopes [30], suggesting that each hyper variable loop may contribute equally to the induction of virus neutralizing antibodies.

## Conclusion

HPV16, 18, 31 and 33 are the four most prevalent HPV high risk types in cervical cancer. So far, HPV31 and 33 are not included in current vaccines. Construction of a multivalent prophylactic vaccine based on chimeric particles should be facilitated by selective combination of simple rather than complex neutralizing epitopes. We have shown here that various surface exposed hyper variable loops of the major capsid protein L1 of HPV33 contribute to the induction of a virus-neutralizing humoral immune response. The complexity of the identified conformational epitopes ranges from rather simple structures, consisting of only one loop, e.g. the BC loop, to epitopes to which several loops contribute. Our data suggest that it should be possible to generate chimeric polyvalent HPV particles that could serve as an intertypic vaccine targeting several HPV types at a time.

## Methods

### Cell lines and antibodies

The osteosarcoma cell line HuTK^-^143B [31] was grown at 37°C in Dulbecco's modified Eagle medium (DMEM) supplemented with 10% fetal calf serum and antibiotics. The human embryonic kidney cell line 293TT [24] was maintained in DMEM/10% FCS with 1% Glutamax I and 1% non-essential amino acids (Invitrogen). Three conformation-dependent, neutralizing mouse monoclonal antibodies, H33.B6 (IgG2a), H33.E12 (IgG2a) and H33.J3 (IgG2b), respectively, with specificity for HPV33 were kindly provided by N. D. Christensen, Hershey, PA. The HPV16-neutralizing mAb H16.56E, was generated by immunization of mice with HPV16 VLPs, and used as previously reported [32,33].

### Construction of hybrid L1 capsomers by site-directed mutagenesis

Type-specific amino acids in hypervariable loops of the HPV33- and HPV16 L1 capsid proteins were identified by CLUSTAL amino acid sequence alignment [34]. For generation of HPV33:16 hybrid virus-like-particles, various loop sequences of the HPV33 L1 capsid protein (BC, DE, FGa, FGb, HI; Fig. 2) were exchanged by the corresponding amino acids of HPV16 by introducing codon-modified sequences from p16L1h [35] into pTM33L1 [12]. HPV16:33 hybrids were generated reciprocally, using the codon-modified pUF3hu16L1 vector and codon-modified loop sequences of HPV33 L1. Overlap extension PCR [36] was used to introduce multiple substitutions simultaneously. Pairs of PAGE-purified mutagenesis primers with 100 % complementarity (Table 1) were purchased from Invitrogen and PCR was carried out using puReTaq Ready-to-go PCR-beads (Amersham Biosciences). In a first step two separate PCR reactions were prepared to generate fragments in forward and reverse orientations, both carrying the desired mutations. Thereby, the reverse mutagenesis primer was used together with an outer forward primer, the forward mutagenesis primer in combination with an outer reverse primer. L1 expression plasmids were used as template and PCR was performed for 40 cycles with denaturation at 95°C for 45 seconds, annealing at 42°C for 1 min and elongation at 72°C for 2 min. PCR fragments generated by these PCRs were purified by agarose gel electrophoresis, followed by Jetsorp gel extraction prior to their use in subsequent reactions. Because of an average overlap of 60 bp between appropriate fragments, these sequences were hybridized by pre-extension PCR [37], in which the 3'overlap of each strand acts as a primer for the extension of the complementary strand. This was done by 2 cycles with denaturation at 95°C for 5 min and annealing at 72°C for 2 min. Resulting products were PCR-amplified by addition of the outer primers of step 1 (conditions: denaturation at 95°C, 45 sec; annealing at 50–56°C, 1 min; elongation at 72°C, 2 min; 35 cycles). Subsequently, the gel-purified mutant L1 amplimers (sized between 800–1900 bp) were cloned into singular restriction sites in the transfer vectors pUF3hu16L1 or pTM33L1 to generate the HPV16/HPV33 or HPV33/HPV16 hybrid L1-constructs. Ligation mixtures were transfected into chemically competent cells of *E. coli *(DH5α). Colonies containing the desired mutations were identified by their newly introduced restriction sites or directly by sequencing. If only one of the two fragments could be generated in the first PCR round, the purified fragment was used in a following PCR as a megaprimer. The fragment was added in excess over the plasmid template and combined with a counter-directed common primer, using the following conditions for a total of 35 cycles: denaturation at 95°C for 45 sec, annealing at 65°C for 1 min, elongation at 72°C for 2 min. Generation of HPV16:33-hybrids with double loop exchanges occurred successively. One loop was introduced by the approach described above. To introduce the second loop, a forward primer was generated using the hybrid L1 as a template. Subsequently, the fragment served as a megaprimer to amplify the complete expression plasmid with high-fidelity Pwo DNA polymerase for 18 cycles (denaturation for 30 sec at 95°C, annealing for 1 min at 50°C, elongation for 14 min at 72°C). The PCR product was then digested with DpnI to eliminate methylated template DNA and the remaining mutant plasmids were expressed in E. coli.

**Table 1:** Codon optimized sequences of mutagenesis primers

**Constructs**	**Sequences for primers (listed 5' to 3')**
**HPV33:BC**	For GGCCATCCATATTTTCCCATCAAGAAGCCCAACAACAACAAATTATTGGTACCC
	Rev GGGTACCAATAATTTGTTGTTGTTGGGCTTCTTGATGGGAAAATATGGATGGCC
**HPV33:DE**	For TTTGATGACATCGAAAACGCCAGCGCCTACGCCGCCAACGCCGGTGCTGATAATAGG
	Rev CCTATTATCAGCACCGGCGTTGGCGGCGTAGGCGCTGGCGTTTTCGATGTCATCAAA
**HPV33:FGa**	For ATGTTTGTAAGACACCTGTTCAACAGGGCCGGCGCCTACGGCGAGAACGTTCCCGATGACCTG
	Rev CAGGTCATCGGGAACGTTCTCGCCGTAGGCGCCGGCCCTGTTGAACAGGTGTCTTACAAACAT
**HPV33:FGb**	For ATTAAAGGTTCAGGAAGCACCGCCAACCTGGCCAGCAGCAACTACTTTCCCACTCCTAGTGG
	Rev CCACTAGGAGTGGGAAAGTAGTTGCTGCTGGCCAGGTTGGCGGTGCTTCCTGAACCTTTAAT
**HPV33:HI**	For AATATGACTTTATGCGCCGCCATCAGCACCAGCGAGACCACCTACAAGAACAACAATTTTAAAGAATATATAAG
	Rev CTTATATATTCTTTAAAATTGTTGTTCTTGTAGGTGGTCTCGCTGGTGCTGATGGCGGCGCATAAAGTCATATT
**HPV16:BC**	For GGCCACCCCTACTTCAGCATCAAGAACCCCACCAACGCCAAGAAGATCCTGGTGCCC
	Rev GGGCACCAGGATCTTCTTGGCGTTGGTGGGGTTCTTGATGCTGAAGTAGGGGTGGCC
**HPV16:DE**	For ACCGGCAACAAGTACCCCGGCCAGCCCGGCGTGGACAACAGGGAGTGCATCAGCATGGAC
	Rev CCTGTTGTCCACGCCGGGCTGGCCGGGGTACTTGTTGCCGGTCTCGGTGTCGTCCAG
**HPV16:FGa**	For ATGTTCGTGAGGCACTTCTTCAACAGGGCCGGCACCCTGGGCGAGGCCGTGCCCGACGACCTG
	Rev CAGGTCGTCGGGCACGGCCTCGCCCAGGGTGCCGGCCCTGTTGAAGAAGTGCCTCACGAACAT
**HPV16:FGb**	For ATCAAGGGCAGCGGCACCACCGCCAGCATCCAGAGCAGCGCCTTCTTCCCCACCCCCAGC
	Rev GCTGGGGGTGGGGAAGAAGGCGCTGCTCTGGATGCTGGCGGTGGTGCCGCTGCCCTTGAT
**HPV16:HI**	For AACATGAGCCTGTGCACCCAGGTGGCCAGCGACAGCACCTACAAGAACGAGAACTTCAAGGAGTACCTG
	Rev CAGGTACTCCTTGAAGTTCTCGTTCTTGTAGGTGCTGTCGCTGGCCACCTGGGTGCACAGGCTCATGTT

### Immunofluorescence analysis

HuTK^- ^cells were grown on glass coverslips overnight, infected with the vaccinia helper virus VTF7-3 for 1 h (MOI of 5) and subsequently transfected using Lipofectamin plus (Invitrogen) and 1 μg transfer plasmid pTM1 carrying wt or mutated HPV33L1 sequences under the control of a T7-promotor. Expression of the pUF3 vector-based wt or hybrid HPV16 L1-constructs occurred by lipofection without any helper viruses. After an incubation period of 10 – 24 h at 37°C cells were fixed with 2 % paraformaldehyde for 20 min at room temperature, permeabilized with 0.1 % Nonident P-40 for 15 min and subsequently blocked in 5 % goat serum dissolved in PBS. Incubations with primary mAbs and secondary Cy2-conjugated Affinipure goat anti-mouse IgG (Jackson Immunoresearch Products) were carried out for 1 h at 37°C. Thereafter, coverslips were washed with PBS several times, stained with 0.2 μg/ml Bis-benzimide trihydrochloride (Hoechst 33342; Sigma) and mounted onto slides by using Fluoprep mounting medium (BioMérieux). Pictures were taken using a Zeiss Axiovert 200 M microscope and a Zeiss Axiocam digital camera. The appropriate Axiovision Software 3.0 was used for merging pictures.

### Preparation of pseudovirions and VLPs

HPV33-VLPs and pseudovirions were produced in HuTK^- ^cells by infection with recombinant vaccinia viruses vac33L1, vac33L2 and helper virus VTF7-3, as described previously [12,38]. For generation of pseudovirions, cells were transfected 24 h prior to infection with a marker plasmid encoding a dimeric green fluorescent protein (GFP), resulting in HPV particles containing the GFP reporter DNA. Forty-four hours post infection VLPs/PsV were extracted from nuclei by sonication in hypotonic buffer supplemented with 0.5% NP-40 and purified by buoyant caesium chloride density gradients. HPV16 pseudovirions were prepared as described previously [24] by co-transfection of 293TT cells with pUF3hu16L1 wt or pUF3hu16/33L1-hybrid plasmids, together with pUF3hu16L2 wt and the pEGFPGFP marker plasmid. Subsequent to incubation at 37°C for 48 h cells were lysed and pseudovirions were purified on an OptiPrep gradient. Thereby, lysis of cells was achieved by adding the non-ionic detergent Brij58 (Sigma) at a final concentration of 0.5 % in DPBS supplemented with 9.5 mM MgCl_2_. Lysates were digested over night at 37°C with 2 U of Benzonase (Sigma) to complete virus maturation [39]. Subsequently the lysate was mixed with a 0.17 volume of 5 M NaCl, clarified by centrifugation at 1500 × g for 10 min, loaded on top of an OptiPrep step gradient (27%/33%/39% OptiPrep in DPBS-800 mM NaCl) and centrifuged for 4h at 234.000 × g. After centrifugation, 250 μl-fractions were collected by bottom puncture of the tubes and 1 μl of each fraction was tested in a pseudovirus infection assay.

### Infection and neutralization assays

Human embryonic kidney 293TT cells were grown overnight in 24-well plates and infected with 1 μl of HPV pseudovirions (PsV) in a total volume of 500 μl DMEM. Cells were grown at 37°C for 72 h and infectious events were monitored by counting cells with green nuclear fluorescence. To perform virus neutralization assays, PsV were bound to cells for 1 h at 4°C, unbound virions were removed and various dilutions of HPV-specific neutralizing antibodies were added to cells in a total volume of 250 μl DMEM. After 1 h at 37°C the culture medium was replaced and incubation was continued for 72 h.

### Heparin-based enzyme-linked immunosorbent assays (Hep-BSA ELISA)

VLP-ELISAs were used to study the interaction of conformationally intact VLPs with heparin and performed as previously described [29,40]. Briefly, polysorb microtiter plates (NUNC, Wiesbaden, Germany) were coated overnight with 100 ng of heparin-BSA/well in phosphate-buffered saline (PBS), washed and subsequently blocked with BSA (50 μg/ml) for 30 minutes. Plates were again washed, 100 μl VLPs (1 μg/ml) were added and incubated for 1 h at 37°C. Unbound particles were eliminated by washing. HPV type-specific antibodies H16.56E, H33.B6, H33.J3 and H33.E12 were added for 1 h at 37°C at the indicated concentrations (1:100 – 1:5000). After washing three times with PBS-Tween 20 (PBS-T), 100 μl horseradish peroxidase-coupled secondary antibodies (goat anti-mouse IgG; 1:10.000 in PBS-T) obtained from Jackson Immunochemicals were added and incubated for additional 30 min at 37°C. Plates were washed and developed with ready to use trimethyl benzidine (KPL). The reaction was stopped after 10 min at 37°C with 100 μl 1N HCl. Absorbance was measured at 450 nm using a Multiscan EX (Thermo Life Sciences).

### Visualization of epitopes by RasMol

The RasMol program is a molecular graphics visualisation tool for macromolecular structures [41]. Localization of amino acids in loops structures of capsid protein L1 from HPV16 or HPV33 was based on the atomic coordinates of the HPV16 major capsid protein L1 [17] and visualized using the PDB file 1DZL in the RasMol program.

## Competing interests

The author(s) declare they have no competing interests with this publication.
